# Dual Antiplatelet Therapy for the Acute Management and Long-term Secondary Prevention of Ischemic Stroke and Transient Ischemic Attack, An Updated Review

**DOI:** 10.3390/jcdd11020048

**Published:** 2024-01-31

**Authors:** Bernard P. L. Chan, Lily Y. H. Wong, Benjamin Y. Q. Tan, Leonard L. L. Yeo, Narayanaswamy Venketasubramanian

**Affiliations:** 1 Division of Neurology, National University Hospital, National University Health System, Singapore 119228, Singapore; lily_wong@nuhs.edu.sg; 2 Division of Neurology, National University Hospital; and Yong Loo Lin School of Medicine, National University of Singapore, Singapore 119228, Singapore; benjamin_yq_tan@nuhs.edu.sg (B.Y.Q.T.); leonardyeoll@gmail.com (L.L.L.Y.); 3 Raffles Neuroscience Centre, Raffles Hospital, Singapore 188770, Singapore; drnvramani@gmail.com

**Keywords:** dual antiplatelet therapy, ischemic stroke, transient ischemic attack, aspirin, clopidogrel, ticagrelor, dipyridamole, cilostazol, combination

## Abstract

To improve the efficacy over antiplatelet monotherapy, dual antiplatelet therapy (DAPT) has been increasingly adopted in the management of non-cardioembolic stroke. For minor ischemic stroke and high-risk transient ischemic attack, the aspirin–clopidogrel combination is now recommended for acute short-term treatment, whereas aspirin–ticagrelor combination may be considered in selected patients, especially those with resistance to clopidogrel. For long-term stroke prevention, aspirin–dipyridamole combination has been used as an alternative to antiplatelet monotherapy, and aspirin or clopidogrel combined with cilostazole may be prescribed for added protection in high-risk patients. In this paper, we review the development of DAPT from a historical perspective and describe the findings from major clinical trials published up until the end of 2023. Using the 2021 American Heart Association guideline for secondary stroke prevention as a basis for our recommendations, we further discuss areas of controversy and more recent developments to provide an updated review for clinicians to consider in their daily practice.

## 1. Introduction

The aspirin–clopidogrel combination has been studied in long-term primary and secondary stroke prevention, acute short-term treatment of non-cardioembolic minor ischemic stroke and transient ischemic attack (TIA), and symptomatic extracranial or intracranial stenosis. More recently, aspirin–ticagrelor combination has been studied in minor stroke and TIA and in a subgroup of these patients who were carriers of CYP2C19 loss-of-function alleles. For long-term secondary prevention, aspirin–dipyridamole combination and either aspirin or clopidogrel combined with cilostazol have been studied. In this paper, we describe major findings from representative randomized clinical trials published up until the end of 2023 and offer our recommendations, which are summarized in Table 1. We follow the 2021 American Heart Association (AHA) guideline for secondary stroke prevention in classifying our recommendations into three levels: Class 1 (recommended), Class 2a (reasonable), and Class 2b (may be considered) [1].

## 2. Aspirin and Clopidogrel in Long-Term Stroke Prevention

Aspirin has remained today the mainstay of secondary stroke prevention [1]. Antiplatelet monotherapy (mainly aspirin) was associated with a 23% reduction in the odds of all strokes in patients with a previous stroke or TIA in a meta-analysis from the Anti-thrombotic Trialists’ Collaboration (published in 2002) [2], but the odds reduction was only 13% in another meta-analysis (1996) [3]. Clopidogrel is associated with a lower risk of gastrointestinal hemorrhage compared to aspirin, but the combined vascular risk was not significantly reduced compared to treatment with aspirin among patients with a previous stroke in the CAPRIE trial (performed in 1992–1996) [4] (A list of Abbreviations for Clinical Studies Cited is provided at the end of the article). More effective antiplatelet therapy in stroke prevention has been keenly awaited.

Encouraged by the positive results in studies with clopidogrel added to aspirin for up to 12 months in patients with acute coronary syndrome [5] or after percutaneous coronary intervention [6], dual antiplatelet therapy (DAPT) with aspirin and clopidogrel (ASA+CLO) has been studied in clinical trials of long-term primary and secondary stroke prevention.

In the CHARISMA trial (2002–2005), 15,603 subjects with multiple vascular risk factors or a history of coronary, cerebrovascular, or peripheral vascular diseases were recruited. Over a median follow-up of 28 months, ASA+CLO was associated with a non-significant reduction in composite vascular outcome (6.8 vs. 7.3%) compared to aspirin alone. However, in the subgroup with a history of vascular diseases (including stroke), a significant reduction in this primary endpoint (6.9 vs. 7.9%) was found [7].

ASA+CLO was further studied in 3020 patients with MRI-proven lacunar infarcts within the preceding 6 months in the SPS3 trial (2003–2011). The trial was terminated early, as ASA+CLO was not associated with any significant reduction in recurrent strokes (2.5 vs. 2.7% per year) but a near doubling of major hemorrhage (2.1 vs. 1.1% per year) compared to aspirin alone [8].

For patients with a high risk of recurrence, the MATCH trial (2000–2003) recruited 7599 patients who had experienced an ischemic stroke or TIA in the previous 3 months and harboring additional risk factors. The ASA+CLO combination was compared to clopidogrel alone. Over 18 months, the primary endpoint of composite vascular outcome was non-significantly reduced from 16.7 to 15.7%, but life-threatening bleeding was significantly increased from 1.3 to 2.6%. However, both life-threatening bleeding and intracranial hemorrhage appeared to increase only after 3 months of DAPT [9]. The other notable feature of the trial is that ASA+CLO was compared to clopidogrel rather than aspirin, possibly leading to more unfavorable hemorrhagic outcomes in the DAPT group compared to antiplatelet monotherapy.

These trials have led to the conclusion that DAPT with ASA+CLO is not recommended for long-term primary or secondary stroke prevention and is contraindicated after lacunar stroke. However, findings from the MATCH trial suggested that acute short-term treatment with ASA+CLO may still be possible in patients with a high risk of early recurrence and a low risk of hemorrhage, such as those with minor ischemic stroke and TIA.

During the same period, ASA+CLO was also studied in patients with non-valvular atrial fibrillation (NVAF). In the ACTIVE-W trial (2003–2005), patients who were warfarin candidates were randomized between ASA+CLO and warfarin, and the study was stopped early due to the clear superiority of warfarin [10]. In the ACTIVE-A trial (2003–2008), patients unsuitable for warfarin were randomized between ASA+CLO and aspirin alone. Over a median follow-up of 3.6 years, DAPT was associated with significant reductions in vascular events (6.8 vs. 7.6% per year) and stroke (2.4 vs. 3.3% per year) but a concurrent increase in major bleeding (2.0 vs. 1.3% per year) [11]. However, with the emergence of non-vitamin K oral anticoagulants (NOAC) and apixaban showing major benefits in stroke reduction but no significant increase in bleeding risk compared to aspirin in similar patients in the AVERROES trial (2007–2010) [12], the role of ASA+CLO in long-term stroke prevention in NVAF has diminished substantially.

## 3. Aspirin and Clopidogrel in Short-Term Treatment of Non-Cardioembolic Minor Stroke and TIA

TIA is a major risk factor for stroke, but the recognition that TIA requires urgent treatment only emerged more recently, when a cohort study performed in 1997–1998 among TIA patients presented to emergency departments in Northern California revealed that 10.5% had a recurrent stroke within 90 days, with half of the strokes having occurred within the first 48 h [13]. In another community-based study performed in 2002–2003 among patients with TIA and minor stroke with National Institute of Health Stroke Scale scores (NIHSS) ≤ 3 in Oxford, the recurrent stroke rate was 8% within 7 days and 17.3% at 3 months [14].

The high early recurrent stroke risk led to the establishment of different TIA treatment protocols around the world, which include same-day TIA clinic, emergency department-based protocol, and hospitalization with a short stay, all aiming to deliver early comprehensive TIA management. Medical therapy including antiplatelet agents and statin are administered immediately after confirmation of the diagnosis of TIA and exclusion of intracranial hemorrhage by urgent neuroimaging. Other medical treatments, dietary advice, and healthy lifestyle counselling are initiated before discharging the patient. DAPT with ASA+CLO is frequently administered to high-risk TIA patients in these protocols.

The EXPRESS study performed in Oxford was a before (2002–2004) and after (2004–2007) comparison of cases with the implementation of a same-day TIA clinic, whereas SOS-TIA was a study performed in 2003–2005 on TIA patients presented to a 24 h clinic at a hospital emergency department in Paris. Both studies reported dramatic reductions in the recurrent 90-day stroke rates when compared with the stroke rates before implementation of the same-day TIA clinic or predicted from the patients’ ABCD2 scores, respectively [15,16]. DAPT with ASA+CLO may have contributed to the beneficial effects in the management of these patients, and this treatment was compared to aspirin alone for 90 days in 392 patients with TIA or minor stroke in the FASTER pilot trial (2003–2007). The ASA+CLO combination led to a non-significant reduction in stroke (7.1 vs. 10.8%) but a 3% symptomatic hemorrhage rate [17]. It was therefore time for definitive trials of acute short-term DAPT with ASA+CLO in patients with TIA and minor ischemic stroke.

The CHANCE trial (2009–2012) randomized 5170 patients in China with minor stroke (NIHSS ≤ 3) or high-risk TIA (ABCD2 ≥ 4) within 24 h of onset to the ASA+CLO combination for 21 days followed by clopidogrel alone from day 22 to day 90 versus aspirin monotherapy for 90 days. The DAPT group had a significant reduction in the 90-day stroke rate (8.2 vs. 11.7%) and no differences in the rates of moderate or severe hemorrhage and hemorrhagic stroke (all 0.3%) compared to the group treated with aspirin alone [18]. POINT was an international trial (2010–2018) that recruited 4881 similar patients with minor stroke or TIA within an earlier time window of 12 h and compared the ASA+CLO combination versus aspirin alone for 90 days. Both the composite vascular endpoint and stroke recurrence at 90 days were significant reduced (5.0 vs. 6.5% and 4.8 vs. 6.4%, respectively), but major hemorrhage was significantly increased with DAPT (0.9 vs. 0.4%) [19].

**Recommendation 1:** Based on results from the CHANCE and POINT trials, the AHA guideline gives a Class 1 recommendation for patients presenting with minor non-cardioembolic ischemic stroke (NIHSS ≤ 3) or high-risk TIA (ABCD2 ≥ 4), DAPT with ASA+CLO should ideally be initiated within 12–24 h but may be considered up to 7 days after symptom onset (based on meta-analyses which included other DAPT studies) and continued for 21 to 90 days, followed by antiplatelet monotherapy [1]. However, several practice points require further clarification:

(a) Loading doses of DAPT: For aspirin, patients in CHANCE received a dose of 75–300 mg on day 1 at the discretion of the treating physician followed by 75 mg daily, whereas in POINT 162 mg daily for 5 days followed by 81 mg daily was recommended. As early aspirin therapy was shown to be associated with substantial benefit in the first 2 weeks after a minor stroke or TIA in a pooled analysis of 12 trials (2016) [20], we recommend a loading dose of 300 mg aspirin if there is no contraindication.

For clopidogrel, a loading dose of 300 mg was given in CHANCE, and 600 mg was given in POINT. There was no increase in major hemorrhage in CHANCE [18], but a small increase was noted early in the POINT trial [19,21]. We, therefore, recommend a clopidogrel loading dose of 300 mg for most patients, but 600 mg may be considered in high-risk patients (e.g., crescendo TIA, severe symptomatic arterial stenosis) after careful consideration of the risks and benefits.

(b) Duration of DAPT: The duration of ASA+CLO therapy was 21 days in CHANCE and 90 days in POINT. However, the benefit of DAPT over aspirin was variable during the 90-day period. A meta-analysis of the FASTER, CHANCE, and POINT trials showed that most of the benefits of DAPT occurred within 10 days of randomization [22]. A patient-level meta-analysis of the CHANCE and POINT trials confirmed that the benefit of DAPT occurred mainly within the first 21 days [23]. Furthermore, a meta-analysis including short-term trials such as CHANCE and POINT and long-term trials such as SPS3 and CHARISMA revealed that short-term DAPT for up to 1 month was associated with significant reductions in ischemic stroke and vascular events; intermediate-term DAPT for up to 3 months was associated not only with significant reductions in ischemic stroke and vascular events but also a significant increase in major bleeding; whereas, long-term DAPT over 3 months was associated with significant increases in both major bleeding and mortality and no significant reductions in ischemic stroke or major vascular events [24].

Based on these findings, we recommend that patients who are indicated for ASA+CLO should generally be prescribed a 21-day course of DAPT, with extensions up to 90 days in selected patients (e.g., progressive or recurrent stroke/TIA, symptomatic severe intracranial stenosis) after careful consideration of the risks and benefits.

(c) TIA with ABCD2 ≤ 3, lacunar stroke, and stroke with NIHSS ≥ 4: The ABCD2 score comprises both clinical features and risk factors for stroke [25]; therefore, an ABCD2 score ≤3 is not only associated with a lower recurrent stroke risk but also increases the likelihood of a TIA mimic. However, other factors associated with a high stroke risk (e.g., symptomatic large artery disease) may be present despite low ABCD2 scores [26]. Therefore, we generally do not recommend DAPT for TIA with ABCD2 scores below 4, unless a high-risk stroke etiology is identified.

Patients with lacunar stroke are contraindicated for long-term ASA+CLO due to their increased bleeding risk, as shown in the SPS3 trial [8]. However, both the CHANCE and POINT trials likely included a large number of lacunar stroke patients with NIHSS ≤ 3, and short-term DAPT for 21 days may be associated with lower bleeding risks compared to long-term treatment. Furthermore, a hospital-based retrospective study from Heidelberg (2010–2017) suggested that a short course of DAPT (given for 5 days in the majority) with ASA+CLO prescribed to lacunar stroke patients with clinical deterioration may result in better outcomes [27]. Lacunar stroke with NIHSS ≤ 3 should, therefore, not be excluded from acute short-term DAPT.

A study from hospitals in the USA found that 47% of minor stroke patients did not receive DAPT, whereas 42.6% of patients with non-minor stroke received DAPT upon discharge [28]. There are some justifiable reasons for prescribing DAPT in strokes with NIHSS > 3, such as in cases of symptomatic large artery atherosclerosis, supported by the recently published INSPIRES trial [29] (see Section 4). We believe that DAPT may be prescribed for strokes with NIHSS > 3 on a case-by-case basis. The factors to consider include a non-disabling stroke despite higher NIHSS, well-controlled blood pressure, absence of neuroimaging features associated with risk of symptomatic intracerebral hemorrhage (e.g., large stroke size, hemorrhagic transformation), together with a high risk of stroke progression or recurrence. Another option to consider is to prescribe DAPT for up to 10 days only to maximize its early beneficial effect but possibly reducing the risk of hemorrhage associated with a longer duration of the therapy [22].

(d) Patients not on aspirin: A meta-analysis of the CHANCE, POINT, and THALES trials revealed that the beneficial effects of DAPT were consistent in patients with or without prior use of aspirin [30]. Therefore, DAPT can be initiated in patients who are not taking any antiplatelet therapy at the onset of stroke or TIA.

## 4. Aspirin and Clopidogrel in Large Artery Atherosclerosis

Large artery atherosclerosis (LAA) is a major etiology of stroke with artery-to-artery embolism as the main stroke mechanism, which can be treated using aggressive antiplatelet therapy. CARESS (published in 2005) and CLAIR (2003–2008) were pilot studies of DAPT with ASA+CLO in patients with symptomatic carotid or intracranial stenosis, respectively, using microembolic signals (MES) on transcranial Doppler (TCD) monitoring as a surrogate outcome. The studies individually revealed significant reductions in MES with DAPT compared to antiplatelet monotherapy, and a pooled analysis showed a significant decrease in recurrent stroke occurrence [31,32].

The WASID study (1999–2003) compared warfarin with aspirin in patients with symptomatic intracranial stenosis of 50–99% severity. While there was no difference in the stroke outcome between the two treatment arms, the recurrent stroke risk at 1 year was particularly high (23%) for patients with intracranial stenosis ≥70% [33,34]. The SAMMPRIS trial (2008–2011) studied this high-risk group of patients with severe symptomatic intracranial stenosis with aggressive medical therapy, with or without stenting. Stenting was associated with a high perioperative stroke rate. However, an aggressive medical therapy that included DAPT with ASA+CLO for 3 months, control of vascular risk factors to stringent targets, and counselling for healthy lifestyle resulted in a 12.2% stroke rate at one year, much lower than that of similar patients in the WASID trial [35].

The recently published INSPIRES trial (2018–23) randomized 6100 patients in China with minor stroke (NIHSS ≤ 5) or high-risk TIA (ABCD2 ≥ 4) with symptomatic extracranial or intracranial LAA within 72 h of onset to the ASA+CLO combination for 21 days followed by clopidogrel from day 22 to day 90 versus aspirin alone for 90 days. Among the participants, 82% had ≥50% symptomatic arterial stenosis, and 87% were randomized at a later time window (compared to CHANCE and POINT) at 24–72 h after symptom onset. DAPT was associated with both a significant decrease in recurrent stroke (7.3 vs. 9.2%) and a significant increase in moderate to severe bleeding (0.9 vs. 0.4%) [29].

**Recommendation 2:** Based on the outcome of the SAMMPRIS study’s medical arm, the AHA guideline gives a Class 2a recommendation that DAPT with ASA+CLO for up to 3 months is reasonable for patients with a recent stroke or TIA with severe symptomatic intracranial stenosis (70–99%) [1]. The INSPIRES trial also supports DAPT for 21 days in patients with 50–69% symptomatic intracranial stenosis or ≥50% extracranial stenosis not scheduled for a revascularization procedure (Class 2a). Other areas that require further discussion include the following:

(a) DAPT beyond 3 months: In clinical practice, we often encounter patients with severe intracranial stenosis who present with recurrent TIA beyond 3 months. Currently, there is no clinical guidance to prolong DAPT, and patients treated medically in SAMMPRIS who continued DAPT beyond 3 months had a numerically lower stroke rate but a higher rate of major bleeding compared to patients who switched to aspirin alone after 3 months, although both results were statistically non-significant [36]. We feel that DAPT with ASA+CLO may be prolonged beyond 3 months in patients with severe intracranial stenosis and recurrent symptoms of cerebral ischemia or hypoperfusion on a case-by-case basis, together with the careful control of risk factors for bleeding and review at 3-monthly intervals.

(b) Symptomatic carotid stenosis: DAPT is commonly prescribed for patients with symptomatic carotid stenosis to decrease the risk of recurrent stroke when awaiting carotid revascularization. While DAPT is indicated for carotid stenting, it may be associated with an increased risk of perioperative bleeding in carotid endarterectomy (CEA). A meta-analysis (2022) of CEA patients showed that, despite a significant reduction in MES on TCD monitoring with DAPT, the reduction in the perioperative stroke rate was not significant, but significant increases in the risks of neck hematoma and re-operation for bleeding were found [37]. We recommend that each center should develop their own protocol in perioperative antiplatelet management for CEA, balancing the risks of recurrent stroke and perioperative bleeding. An option to consider is to give a loading dose of DAPT while continuing maintenance antiplatelet monotherapy to maximize early reduction in stroke risk, while decreasing the risk of perioperative hemorrhage when CEA is performed after a few days.

## 5. Aspirin and Clopidogrel Instead of Intravenous Thrombolysis in Minor Non-Disabling Stroke within 4.5 h of Onset

The PRISMS trial (2014–2017) enrolled 313 patients with non-disabling stroke (NIHSS 0–5) and compared intravenous alteplase with 325 mg aspirin within 3 h of onset. Alteplase treatment was associated with no improvement in the outcome at 90 days and a slight increase in symptomatic intracranial hemorrhage (sICH) [38]. The ARAMIS trial (2018–2022) recruited 760 patients with minor stroke (NIHSS ≤ 5) from China and randomized them to DAPT for 12 days (with 300 mg clopidogrel loading) or alteplase, followed by routine care. Non-inferiority at 90 days for excellent outcome was found for DAPT [39]. Furthermore, a retrospective study from the Austrian stroke units (2018–2021) among acute stroke patients with NIHSS ≤ 3 found that alteplase treatment was associated with more sICH and early neurological deterioration compared to DAPT, after propensity score matching [40].

**Recommendation 3:** This topic has not yet been reviewed in the AHA guideline, but we believe that DAPT instead of intravenous thrombolysis is a reasonable treatment option in non-disabling stroke with NIHSS ≤ 5 when high-risk features of stroke progression such as large vessel occlusion or capsular warning syndrome are absent (Class 2a). Consideration may be given to the use of higher loading doses with 300 mg aspirin and 600 mg clopidogrel in selected patients to facilitate early antiplatelet action.

## 6. Aspirin and Ticagrelor in Short-Term Treatment of Non-Cardioembolic Minor Stroke and TIA

Ticagrelor (TIC) is a more potent and direct-acting P2Y12 inhibitor compared to clopidogrel, which requires metabolic activation. Ticagrelor was compared to aspirin in patients with minor stroke (NIHSS ≤ 5) or high-risk TIA (ABCD2 ≥ 4 or symptomatic extracranial/intracranial stenosis) within 24 h of onset in the SOCRATES trial (2014–2016) with treatment continued for 90 days. Non-significant reductions in the primary composite vascular endpoint (6.7 vs. 7.5%) and ischemic stroke (5.8 vs. 6.7%) were noted. In a subgroup analysis, the patients already on aspirin showed a larger reduction in the primary outcome (6.5 vs. 8.4%), suggesting that DAPT with aspirin and ticagrelor (ASA+TIC) may have a better efficacy [41].

In the THALES trial (2018–2019), 11,016 patients with minor stroke (NIHSS ≤ 5) or high-risk TIA (ABCD2 ≥ 6 or symptomatic extra/intracranial stenosis) were randomized to receive ASA+TIC versus aspirin alone for 30 days. Stroke and death within 30 days were significantly reduced (5.5 vs. 6.6%), but severe bleeding was significantly increased (0.5 vs. 0.1%) with DAPT [42]. In a subgroup analysis, the patients with ipsilateral LAA (defined as stenosis ≥30%) had a higher stroke risk and a higher reduction in their stroke and death rate with DAPT (8.1 vs. 10.9%), but there was no statistically significant interaction between the treatment and LAA status [43].

**Recommendation 4:** Based on the findings of THALES and its LAA subgroup analysis, the AHA guideline states that DAPT with ASA+TIC may be considered in patients who presented within 24 h with a minor stroke (NIHSS ≤ 5) or high risk TIA (ABCD2 ≥ 6), with or without ipsilateral large artery stenosis ≥30% (Class 2b) [1].

Since ASA+CLO appears to be associated with a similar recurrent stroke reduction but a lower risk of major hemorrhage in the CHANCE and POINT trials compared to ASA+TIC in the THALES trial, we believe that DAPT with ASA+CLO for 21 days is the first-choice therapy among minor stroke and high-risk TIA patients who satisfy both the CHANCE and POINT inclusion criteria (NIHSS ≤ 3, ABCD2 ≥ 4) (Class 1). DAPT with ASA+CLO for 21 days may also be considered in patients outside the CHANCE–POINT but within the THALES inclusion criteria (NIHSS 4–5, or any TIA with symptomatic extra/intracranial stenosis) after the recent publication of the INSPIRES study, which included some patients in this category (Class 2b). DAPT with ASA+TIC, however, may be considered for patients with recurrent strokes while on clopidogrel or carrying a CYP2C19 allele associated with clopidogrel resistance (Class 2b) (see Section 7).

## 7. Aspirin and Ticagrelor in Short-Term Treatment of Non-Cardioembolic Minor Stroke and TIA in Carriers of CYP2C19 Loss-of-Function Alleles

Clopidogrel is a pro-drug, and it is converted into an active drug by the liver cytochrome P450 isoenzymes. Carriers of the CYP2C19 loss-of-function (LOF) alleles have a decreased conversion of clopidogrel to active drug and, thus, may have reduced benefits with clopidogrel therapy. In a sub-study of the CHANCE trial, 2933 Chinese patients were genotyped, and 58.8% of them had LOF alleles. These carriers had no significant reduction in recurrent stroke rate from DAPT compared to aspirin monotherapy (9.4 vs. 10.8%), whereas a significant reduction was seen in the non-carriers (6.7 vs. 12.4%) [44].

The PRINCE trial (2015–2017) was a phase-II study conducted in China that compared DAPT with ticagrelor versus clopidogrel in reducing high platelet reactivity after 90 days of treatment, with both groups being treated with aspirin for the first 21 days. A total of 650 patients with minor stroke or TIA were recruited within 24 h, and 57.5% of them were LOF allele carriers. High platelet reactivity was observed in fewer ticagrelor-treated patients compared to the clopidogrel-treated patients at 90 days in all the participants (12.5 vs. 29.7%). However, this difference was only marginally increased among the CYP2C19 LOF allele carriers (10.8 vs. 35.4%) [45].

CHANCE-2 (2019–2021) was a randomized trial that compared DAPT with ticagrelor versus clopidogrel plus aspirin in carriers of the CYP2C19 LOF alleles. DAPT was prescribed for 21 days, followed by ticagrelor or clopidogrel monotherapy for up to 90 days. A total of 11,255 Chinese patients with minor stroke (NIHSS ≤ 3) or high-risk TIA (ABCD2 ≥ 4) were screened, and 6412 of them, who were confirmed carriers, were recruited. The average turnaround time for the point-of-care genotyping was 85 min, making the study possible when urgent antiplatelet treatment was required in these patients. The 90-day stroke rate was significantly reduced in the ASA+TIC group compared to the ASA+CLO group (6.0 vs. 7.6%), with no increase in moderate or severe bleeding (0.3% in both) [46].

**Recommendation 5:** This topic has not yet been reviewed in the AHA guideline. We believe that CYP2C19 genotyping is reasonable for the selection of LOF allele carriers to receive DAPT with ASA+TIC in patients with minor stroke or high-risk TIA when they have a history of recurrent events while on clopidogrel (Class 2a). This treatment may also be considered in all minor stroke or high-risk TIA patients when genotyping can be obtained in an economical and expedited manner before treatment (Class 2b).

We believe that more studies are required in this area to clarify issues including which LOF alleles are the most predictive of clopidogrel resistance and clinical outcome, whether genotyping provides any advantage over the more routinely performed platelet reactivity tests, whether selective treatment after genotyping is better than treating all patients with ASA+TIC without genotyping, and the unexpected finding that this treatment option was associated with more benefits in lower-risk strokes due to small artery disease compared to higher-risk strokes due to LAA in a subgroup analysis of CHANCE-2 [47].

## 8. Aspirin and Dipyridamole in Long-Term Secondary Stroke Prevention

Dipyridamole is an inhibitor of platelet cAMP-phosphodiesterase (PDE) and has an additional vasodilation effect. The aspirin and dipyridamole combination (ASA+DIP) has been proven a safe therapy in the long-term prevention of non-cardioembolic stroke. However, some patients are unable to tolerate dipyridamole due to headaches, dizziness, and gastrointestinal upset.

The ESPS trial (1979–1985) studied aspirin with short-acting dipyridamole 75 mg three times daily and showed a 37.5% relative risk reduction in stroke compared to placebo [48]. ESPS-2 (1989–1995) compared treatments with aspirin 50 mg daily, extended-release dipyridamole 200 mg twice daily, both (ASA+DIP), and placebo. The effects in stroke prevention were additive for each antiplatelet treatment, and the ASA+DIP combination had a 23% relative risk reduction in stroke recurrence compared to aspirin alone [49]. The benefit of the ASA+DIP combination over aspirin monotherapy was confirmed in the ESPRIT trial (1997–2005), when the hazard of the primary composite outcome of vascular events was reduced by 20% [50]. However, this benefit was observed only after 6 months of therapy in ESPS-2 and after 2 years in ESPRIT in the ASA+DIP groups.

ASA+DIP was compared to clopidogrel in the PRoFESS trial (2003–2008) in 20,322 patients who were followed up for a mean of 2.5 years. The recurrent stroke rates were similar (9% vs. 8.8%), but major hemorrhage was increased in the ASA+DIP group (4.1 vs. 3.6%). Adverse effects resulting in treatment discontinuation occurred more often with ASA+DIP (16.4 vs. 10.6%), but a 5.9% discontinuation rate due to headache was lower than in previous studies, likely due to a protocol of slower dose escalation and the use of simple analgesics when required in the ASA+DIP group in this trial [51].

**Recommendation 6:** The AHA guideline gives a Class 1 recommendation for using aspirin, clopidogrel, or ASA+DIP (the extended-release form) in secondary stroke prevention, but it does not prefer one option over another [1]. This recommendation is based on the findings that no significant differences were found between aspirin and clopidogrel in the CAPRIE trial and between ASA+DIP and clopidogrel in the PRoFESS trial.

Many clinicians, including us, take a slightly different view. We regard the benefits of ASA+DIP over aspirin monotherapy shown in the ESPS-2 and ESPRIT trials to be robust, and it is reasonable to consider either ASA+DIP or clopidogrel monotherapy in patients who fail aspirin therapy or require added protection (Class 2a). Clopidogrel has advantages over ASA+DIP with a simpler dosing regime and fewer adverse effects, but ASA+DIP will be useful in many patients, especially in those who fail clopidogrel monotherapy. In our practice where extended-release dipyridamole is not available, we use aspirin with short-acting dipyridamole 75 mg three times daily, as this combination was associated with a similar stroke risk reduction in the ESPS trial to the ASA+DIP (extended-release form) arm in the ESPS-2 trial (38 vs. 37%) compared to the placebo [48,49].

## 9. Aspirin or Clopidogrel with Cilostazol in Long-Term Secondary Stroke Prevention

As a specific inhibitor of platelet cAMP-PDE3, cilostazol may have some pharmacological advantages over dipyridamole in antiplatelet and vasodilatation effects, and it has been approved in East Asian countries for secondary stroke prevention. Compared to aspirin, cilostazol has a similar or possibly slightly better efficacy, but bleeding complications are significantly reduced, making it an excellent candidate for combination antiplatelet therapy in long-term use [52].

Similar to dipyridamole, common adverse effects with cilostazol use include headaches, dizziness, and gastrointestinal upset. Therefore, slow dose escalation and symptomatic treatment of headaches when required are also recommended when initiating cilostazol. The ADS trial (2011–2017) performed in Japan among acute non-cardioembolic stroke patients showed that the aspirin and cilostazole combination (ASA+CIL) given within 48 h of stroke onset did not result in any decrease in short-term stroke progression or recurrence within 14 days nor functional improvement at 3 months over aspirin alone [53].

The CSPS.com trial (2013–2018) recruited MRI-proven stroke patients from Japan with high-risk features, defined as having two or more vascular risk factors or extracranial or intracranial stenosis ≥50%. They were randomized to cilostazol added to either aspirin or clopidogrel (ASA/CLO + CIL) compared to aspirin or clopidogrel monotherapy. The trial was stopped after recruiting 1884 of the projected sample size of 4000 patients due to slow recruitment. Among the participants, 29% had intracranial stenosis, and 13% had extracranial stenosis. Combination antiplatelet therapy with cilostazol resulted in a lower rate of ischemic stroke (2.2 vs. 4.5% per year) and no significant differences in hemorrhagic events compared to antiplatelet monotherapy. Ten percent of the patients in the combination therapy group had to stop treatment because of headaches [54].

**Recommendation 7:** The AHA guideline only gives a Class 2b recommendation restricted to patients with 50–99% symptomatic intracranial stenosis that cilostazol, in addition to aspirin or clopidogrel, may be considered for secondary stroke prevention [1], after reviewing CSPS.com and a few smaller trials in patients with intracranial stenosis. The lower class of recommendation is likely due to the non-blinded nature and modest sample size of CSPS.com and the absence of data outside East Asia.

We agree that more data are required, especially from non-Asian populations. However, we are encouraged by the efficacy of cilostazol combination therapy among the patients with intracranial stenosis and lacunar stroke in the CSPS.com trial [55,56] and the consistent finding of no increases in major bleeding across studies [52]. We feel that the addition of cilostazol to either aspirin or clopidogrel for long-term secondary stroke prevention may be considered in all high-risk patients that fulfill the CSPS.com inclusion criteria (Class 2b), and that it is a reasonable treatment option in East Asian countries where more experience and trial data of this therapy are available (Class 2a).

## 10. Discussion

DAPT with different combinations have become important antiplatelet regimes to provide added protection in the secondary prevention of non-cardioembolic stroke and TIA over antiplatelet monotherapy; however, they have continued to be underused in routine clinical practice [28]. The ASA+CLO combination is strongly recommended for the acute short-term treatment of minor stroke and high-risk TIA, but the different treatment protocols of the CHANCE and POINT trials, the overlap of patient groups recruited to ASA+CLO and ASA+TIC trials, and the misunderstanding that antiplatelet-naive patients should not be treated with DAPT are among the reasons for clinical underuse. For long-term secondary prevention, there has been controversy in the efficacy of ASA+DIP and limited experience with ASA/CLO + CIL, but we believe that both ASA+DIP and clopidogrel monotherapy are reasonable antiplatelet options over aspirin monotherapy, and ASA/CLO + CIL combination may be considered in high-risk patients.

We hope that our review could provide support and confidence for the use of different DAPT regimes among clinicians in both acute short-term therapy for minor stroke and high-risk TIA and long-term secondary stroke prevention. We summarize our recommendations in Table 1.

## 11. Conclusions

In acute non-disabling ischemic strokes within 4.5 h of symptom onset, the aspirin and clopidogrel combination is a reasonable treatment option instead of intravenous thrombolysis. In non-cardioembolic minor ischemic stroke and high-risk TIA, a short-term therapy of aspirin–clopidogrel combination for 21 days is recommended, but it is reasonable to extend this treatment to 3 months in symptomatic severe intracranial stenosis. Aspirin–ticagrelor combination for 3–4 weeks may be considered in selected patients with minor stroke and high-risk TIA, especially in those carrying a CYPC19 loss-of function allele associated with clopidogrel resistance. For the long-term secondary prevention of non-cardioembolic stroke, aspirin–dipyridamole combination is a reasonable alternative to clopidogrel monotherapy in patients who require added protection over aspirin alone, and aspirin or clopidogrel combined with cilostazol may be considered in high-risk patients.

## Figures and Tables

**Table 1:** DAPT options for different ischemic stroke presentations.

Presentation	DAPT Regime	Selection Criteria	Recommendation
Acute Ischemic Stroke Treatment			
Acute ischemic stroke ≤4.5 h of onset	ASA 300 mg + CLO 300 mg (600 mg in selected patients), instead of IV thrombolysis	NIHSS ≤ 5, Non-disabling, No LVO or capsular warning syndrome	Class 2a, reasonable
Short-Term Treatment for Non-Cardioembolic Ischemic Stroke or TIA up to 3 Months			
Minor Stroke or High-Risk TIA ≤ 24 h of onset (may be considered up to 7 days)	ASA 300 mg + CLO 300 mg (600 mg in selected patients), followed by ASA+CLO for 21 days (up to 90 days in selected patients)	NIHSS ≤ 3, ABCD2 ≥ 4	Class 1, recommended
		NIHSS 4–5, ABCD2 ≤ 3 with symptomatic extra/intracranial stenosis	Class 2b, may be considered
	ASA 300 mg + TIC 180 mg, followed by ASA+TIC for 30 days	NIHSS ≤ 5, ABCD2 ≥ 6 or symptomatic extra/intracranial stenosis	Class 2b, may be considered
Minor Stroke or High-Risk TIA ≤ 24 h of onset with CYP2C19 LOF allele	ASA 300 mg + TIC 180 mg followed by ASA+TIG for 21 days, and TIC alone from day 22 to 90	NIHSS ≤ 3, ABCD2 ≥ 4	Class 2b, may be considered
		NIHSS ≤ 3, ABCD2 ≥ 4, and history of recurrent stroke/TIA while on CLO	Class 2a, reasonable
Short-Term Treatment for Ischemic Stroke or TIA secondary to Large Artery Atherosclerosis up to 3 Months			
Minor Stroke or High-Risk TIA ≤ 72 h with symptomatic atherosclerosis	ASA 300 mg + CLO 300 mg, followed by ASA+CLO for 21 days	Symptomatic stenosis (≥50%) of an extracranial or intracranial artery	Class 2a, reasonable
Non-disabling stroke or TIA ≤ 30 days with severe intracranial stenosis	ASA+CLO for 90 days, with loading doses given when appropriate	Symptomatic severe stenosis (70–99%) of a major intracranial artery	Class 2a, reasonable
Long-Term Secondary Prevention			
Non-Cardioembolic Ischemic Stroke or TIA	ASA + DIP-ER	Insufficient protection with ASA alone, alternative to CLO monotherapy	Class 2a, reasonable
	ASA/CLO + CIL	Extra/intracranial major artery stenosis ≥50%, or 2 of (age ≥ 65, HT, DM, CKD, PVD, previous IS, IHD, current smoking)	Class 2b, may be considered
	ASA+CLO	Non-cardioembolic stroke	Not recommended
		Lacunar stroke	Contra-indicated
Cardioembolic stroke or TIA with NVAF		Cardioembolic stroke or TIA with NVAF unsuitable for VKA or NOAC	Class 2b, may be considered

ABCD2: (age, blood pressure, clinical features, duration of symptoms, and diabetes) score; ASA: aspirin; CKD: chronic kidney disease; CIL: cilostazole; CLO: clopidogrel; DIL-ER: dipyridamole extended release; DM: diabetes mellitus; HT: hypertension; IHD: ischemic heart disease; IS: ischemic stroke; LOF: loss of function; LVO: large vessel occlusion; NIHSS: National Institute of Health Stroke Scale score; NOAC: non-vitamin K oral anticoagulant; NVAF: non-valvular atrial fibrillation; PVD: peripheral vascular disease; TIC: ticagrelor; and VKA: vitamin K antagonist.

## Data Availability

No new data were created or analyzed in this study.

## Abbreviations for Clinical Studies Cited

**ACTIVE-A ^11^**	Atrial Fibrillation Clopidogrel Trial with Irbesartan for Prevention of Vascular Events—Aspirin
**ACTIVE-W ^10^**	Atrial Fibrillation Clopidogrel Trial with Irbesartan for Prevention of Vascular Events—Warfarin
**ADS ^53^**	Acute Aspirin Plus Cilostazol Dual Therapy for Non-Cardiogenic Stroke Patients Within 48 h of Symptom Onset
**ARAMIS ^39^**	Antiplatelet vs. R-tPA for Acute Mild Ischemic Stroke
**AVERROES ^12^**	Apixaban Versus Acetylsalicylic Acid to Prevent Stroke in Atrial Fibrillation Patients Who Have Failed or Are Unsuitable for Vitamin K Antagonist Treatment
**CAPRIE ^4^**	Clopidogrel versus Aspirin in Patients at Risk of Ischemic Events
**CARESS ^31^**	Clopidogrel and Aspirin for Reduction in Emboli in Symptomatic Carotid Stenosis
**CHANCE ^18^**	Clopidogrel in High-Risk Patients with Acute Non-disabling Cerebrovascular Events
**CHANCE-2 ^46^**	Ticagrelor or Clopidogrel with Aspirin in High-Risk Patients with Acute Non-disabling Cerebrovascular Events II
**CHARISMA ^7^**	Clopidogrel for High Atherothrombotic Risk and Ischemic Stabilization, Management, and Avoidance
**CLAIR ^32^**	Clopidogrel plus Aspirin for Infarction Reduction in Acute Stroke or Transient Ischemic Attack Patients with Large Artery Stenosis and Microembolic Signals
**CSPS.com ^54^**	Cilostazol Stroke Prevention Study for Antiplatelet Combination
**ESPRIT ^50^**	European/Australasian Stroke Prevention in Reversible Ischemia Trial
**ESPS ^48^**	European Stroke Prevention Study
**ESPS-2 ^49^**	European Stroke Prevention Study 2
**EXPRESS ^15^**	Early use of Existing Preventive Strategies for Stroke
**FASTER ^17^**	Fast Assessment of Stroke and TIA to Prevent Early Recurrence
**INSPIRES ^29^**	Intensive Statin and Antiplatelet Therapy for Acute HighRisk Intracranial or Extracranial Atherosclerosis
**MATCH ^9^**	Management of Atherothrombosis with Clopidogrel in High-risk Patients
**POINT ^19^**	Platelet-Oriented Inhibition in New TIA and Minor Ischemic Stroke
**PRINCE ^45^**	Platelet Reactivity in Acute Stroke or Transient Ischemic Attack
**PRISMS ^38^**	Potential of rtPA for Ischemic Strokes with Mild Symptoms
**PRoFESS ^51^**	Prevention Regimen for Effectively Avoiding Second Strokes
**SAMMPRIS ^35^**	Stenting and Aggressive Medical Management for Preventing Recurrent Stroke in Intracranial Stenosis
**SOCRATES ^41^**	Acute Stroke or Transient Ischemic Attack Treated with Aspirin or Ticagrelor and Patient Outcomes
**SPS3 ^8^**	Secondary Prevention of Small Subcortical Strokes
**THALES ^42^**	Acute Stroke or Transient Ischemic Attack Treated with Ticagrelor and Acetylsalicylic Acid for Prevention of Stroke and Death
**WASID ^33^**	Warfarin–Aspirin Symptomatic Intracranial Disease Trial
